# Supplementary material for: The Alkylating Agent Methyl Methanesulfonate Triggers Lipid Alterations at the Inner Nuclear Membrane That Are Independent from Its DNA-Damaging Ability
Source: Int J Mol Sci. 2021 Jul 12;22(14):7461. doi: 10.3390/ijms22147461 (PMC8305661; doi:10.3390/ijms22147461)
Supplement: Supplementary file 1 [file ijms-22-07461-s001.zip › ijms-1262831-supplementary.pdf]

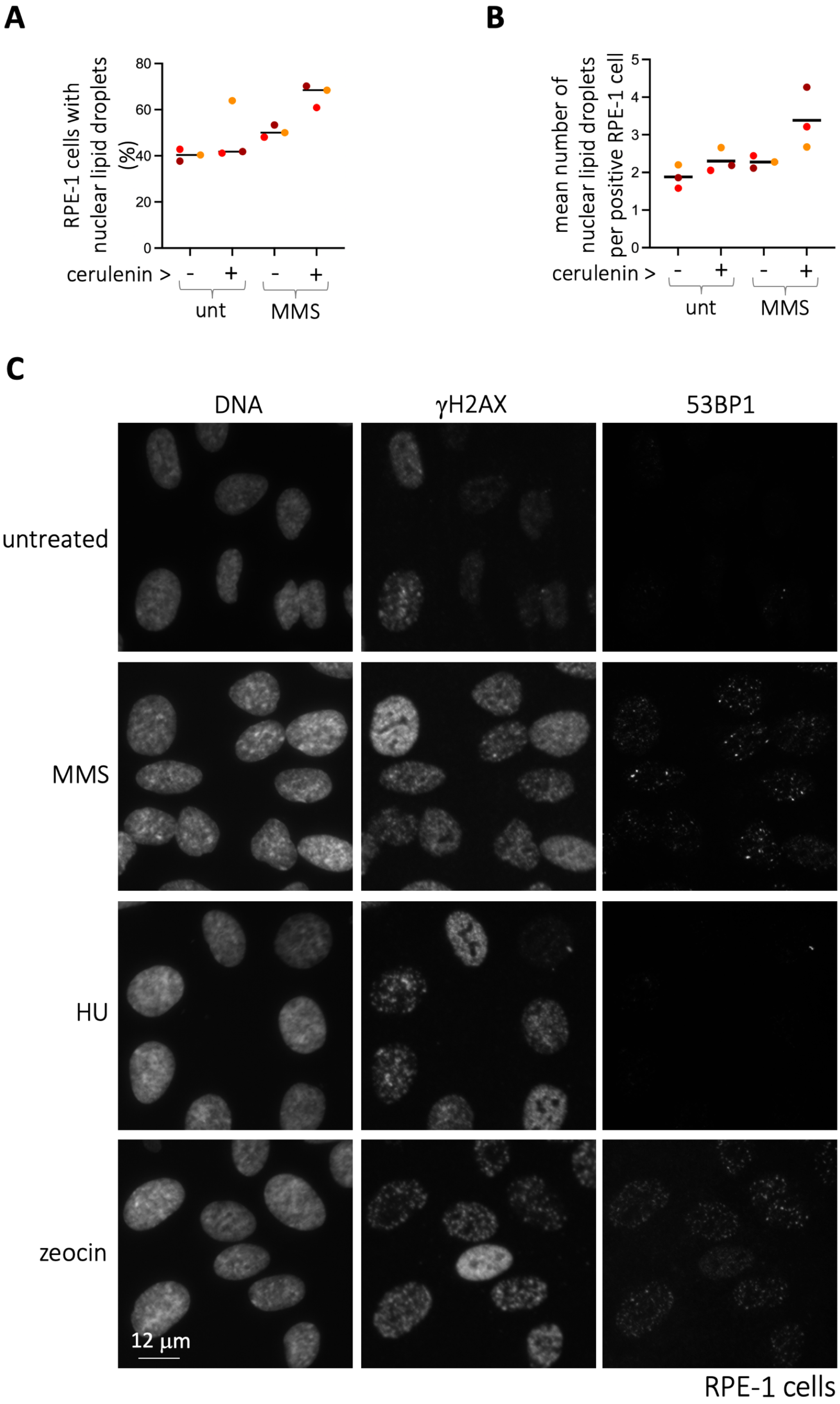

Figure S1. Additional information for Figure 1

(A) Graph showing the percentage of RPE-1 cells displaying at least one nLD under the indicated conditions (details as in Figure 1). Each dot is the mean value of one independent experiment. Dots belonging to a same experiment are plotted in a similar colour. The horizontal black line is the mean of the means. **(B)** Details as in (A) but points represent the mean number of nLD per cell when considering only the positive ones. **(C)** Immunofluorescence was performed on fixed RPE-1 cells treated with the mentioned agents (details as in Figure 1) to detect 53BP1 and γH2AX foci.

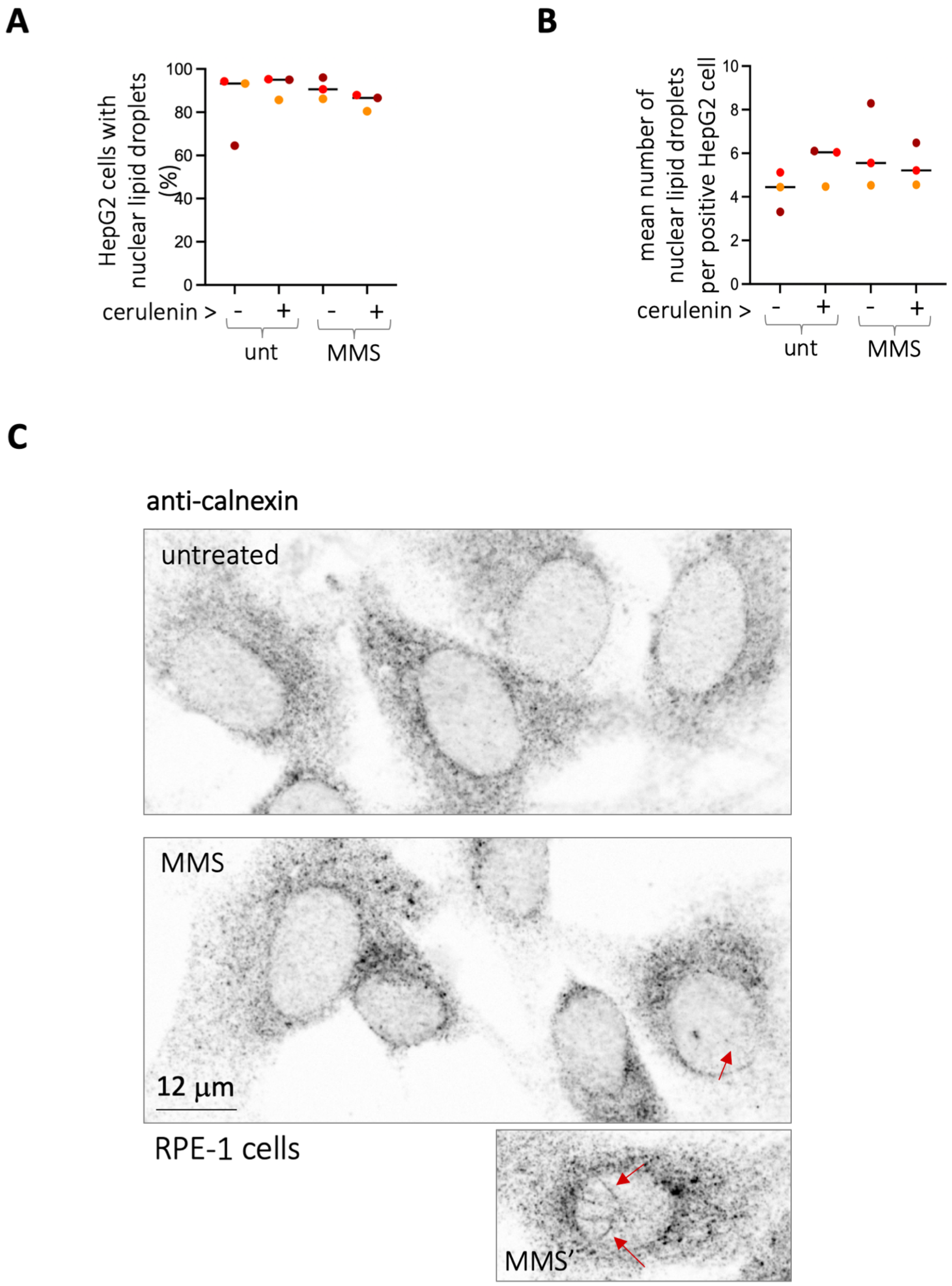

Figure S2. Additional information for Figures 2 and 3

(A) Graph showing the percentage of HepG2 cells displaying at least one nLD under the indicated conditions (details as in Figure 2). Each dot is the mean value of one independent experiment. Dots belonging to a same experiment are plotted in a similar colour. The horizontal black line is the mean of the means. **(B)** Details as in (A) but points represent the mean number of nLD per cell when considering only the positive ones. **(C)** Immunofluorescence was performed on fixed RPE-1 cells treated as indicated (details as in Figure 3) to detect calnexin. Eventual, very rare situations in which calnexin-positive structures dive into the nucleoplasm are marked by red arrows.
